# Practical Notes on Diagnosis and Treatment

**Published:** 1907-02-02

**Authors:** 


### Practical Notes on Diagnosis and Treatment.

**Dyspepsia and Flatulence.**

Sub-gallate of Bismuth (dermatol) has been found, in ten-grain doses after meals, almost a specific in cases of purely functional dyspepsia and flatulence.—*Dr. Flint.*

**Nutrient Enema.**

Three ounces of beef-tea, prepared without salt, are to be added to an ounce of hot water. The enema must be comfortably warm, about 100° F.; if too cold or too hot it will not be retained

**Counter Irritation in Chronic Bronchitis.**

Some stimulating liniment should be rubbed thoroughly into the chest night and morning. What I generally use is the turpentine and acetic acid liniment of the Pharmacopœia. The benefit is usually most marked where there is emphysema, and in these cases a croton oil liniment is often most useful.—*Dr. T. H. Green.*

**Drugs in Acute Bronchitis.**

At the onset of acute bronchitis ipecacuanha is the most useful drug. Do not be afraid of it—ten or fifteen minims every two hours for the first day or two of the illness, or until the expectoration is free and the skin perspiring. Tartar emetic might be used much more frequently than it is, and apomorphine is also a useful drug.—*Dr. T. H. Green.*

**Diarrhœa in Enteric Fever.**

If the diarrhœa is so great as to be exhausting to the patient's strength, and the patient is on an almost exclusively milk diet, you should first examine the stools for the presence of milk-curds; and if these are found to be present, alter the diet to beef-tea and to milk well diluted with lime or soda (not merely carbonated) water, and give small doses of bismuth. Never be in too great a hurry to adopt any powerful astringent treatment.—*Dr. Curnow.*

**Angina Pectoris in Gouty Patients.**

Angina in the gouty requires our most penetrating attention, and there are those who require, even it may be in excess of their usual habit, perhaps one of those very things that in the unwisdom of routine we might be inclined to cut them off. Certainly there are some to whom port wine, champagne, or cream or butter or sweet stuff—to name the reputedly most obnoxious articles that occur to me at the moment—will, each in its place and opportunely, bring healing on its wings.—*Dr. Goodhart.*

**Treatment of Iritis.**

In the early stage of iritis, if the pupil can be fully dilated and maintained in dilatation, the inflammation will die out harmlessly; and hence in this early stage it frequently happens that no other treatment than the application of atropine is required. On the other hand, when the effusion has had time to acquire firmness, and when the dilator muscle is weakened by inflammation, it is very possible that the adhesions may resist atropine. Our sheet anchor then is mercury.—*Mr. Brudenell Carter.*

**Hæmoglobinuria and Raynaud's Disease.**

It is a well-known fact that these two conditions may occur in one and the same patient.

**Purulent Expectoration in Bronchitis.**

When the sputum in chronic bronchitis is copious and puriform, mineral acids with strychnine or quinine are often of great service—more so than the gum resins.—*Dr. T. H. Green.*

**Ærated Milk.**

Milk charged with carbonic acid gas may be used for patients with feeble digestive power and where a change from ordinary milk is required. It is both more palatable and more refreshing than ordinary cow's milk.

**Tabes Dorsalis.**

To relieve the lightning pains of tabes dorsalis, phenacetin in 8 grain doses may be given every half-hour for four doses. It is well to keep the patient lying down during, and for some time after, the administration of the drug.

**Fermentative Dyspepsia.**

The following is a prescription of the late Dr. B. W. Richardson:—Creosote ♏xij, proof spirit ʒss, benzoate of ammonium ʒij, glycerine ʒvj, infusion of cloves to ℥vj. Dose: A tablespoonful in a glass of water after food.

**Bronchitis with Difficult Secretion.**

The following is useful in that form of chronic bronchitis where there is difficulty in coughing up the secretions:—Oil of turpentine ʒiij, mucilage of acacia q.s., cinnamon water ℥j, water to ℥vj. Dose: A tablespoonful every four hours.

**The Nasal Mucous Membrane in Anæmia.**

The mildest of the conditions in which there is dryness of the nasal mucous membrane is simple anæmia, where we have collapse of the erectile tissue, insufficient supply of blood to the mucous glands of the inferior turbinated body, and consequent diminution of the secretion. This condition is not a disease, but simply a symptom of general anæmia.—*Dr. Greville Macdonald.*

**Injury to the Back.**

Never treat lightly a case of injury to the back, no matter how trivial the cause may be. If there is the least indication of interruption of bladder power, or if there is ever so little tingling about the hands, feet, or trunk, such a case may prove disastrous, and should be treated at once as being serious.—*Sir Wm. H. Bennett.*

**Furunculosis of the External Auditory Meatus.**

In the early stages of the inflammation instil frequently a solution of boracic acid in equal parts of rectified spirit and water, gradually diminishing the proportion of water until the patient can bear the application of the undiluted spirit. Apply hot fomentations or boracic poultices. Also gently insert elongated plugs of cotton-wool soaked in glycerine to which a 20-per-cent. solution of cocaine has been added. As soon as there is the slightest indication of the formation of matter a free incision should be made.—*Mr. H. E. Cumberbatch.*

